# Phase I/II study to assess the clinical pharmacology and safety of single ascending and multiple subcutaneous doses of PF-06881894 in women with non-distantly metastatic breast cancer

**DOI:** 10.1007/s00280-021-04355-6

**Published:** 2021-10-07

**Authors:** Hsuan-Ming Yao, Sarah Ruta Jones, Serafin Morales, Shahrzad Moosavi, Jeffrey Zhang, Amy Freyman, Faith D. Ottery

**Affiliations:** 1 Pfizer Inc, Lake Forest, IL USA; 2 Clinical Development and Operations, Pfizer Inc, Collegeville, PA USA; 3 Hospital Universitario Arnau de Vilanova, Lleida, Spain; 4 Pfizer Inc, New York, NY USA; 5 Pfizer Inc, Cambridge, MA USA; 6 Ottery & Associates LLC, Deerfield, IL USA

**Keywords:** Biosimilar, Breast cancer, Chemotherapy, Neutropenia, Pegfilgrastim, Myelosuppression

## Abstract

**Purpose:**

To evaluate the pharmacodynamics (PD), pharmacokinetics (PK), and safety of single and multiple doses of PF-06881894 (pegfilgrastim-apgf; Nyvepria^™^), a biosimilar to reference pegfilgrastim (Neulasta^®^), in women with non-distantly metastatic breast cancer.

**Methods:**

In Phase I (Cycle 0) of this Phase I/II study, the PD response (absolute neutrophil count [ANC]; CD34 + count), PK profile, and safety of a single 3- or 6-mg subcutaneous dose of PF-06881894 were assessed in chemotherapy-naïve patients before definitive breast surgery. In Phase II (Cycles 1–4), the PD response (duration of severe neutropenia [DSN, Cycle 1], ANC [Cycles 1 and 4]) and PK profile (Cycles 1 and 4) of single and multiple 6-mg doses of PF-06881894 concomitant with chemotherapy and after definitive breast surgery were assessed.

**Results:**

Twenty-five patients (mean age 59 years) were enrolled (Cycle 0, *n* = 12; Cycles 1–4, *n* = 13). In Cycle 0, PD responses and PK values were lower with 3-mg versus 6-mg PF-06881894. In Cycles 1 and 4, mean DSN was 0.667 days after single or multiple 6-mg doses of PF-06881894, respectively. In Cycle 4 versus Cycle 1, PD responses were more robust; PK values (mean area under the curve, maximum concentration) were lower; and clearance values were higher. The safety profile of PF-06881894 was similar to that for reference pegfilgrastim.

**Conclusion:**

PF-06881894 as a single 3- or 6-mg dose prior to definitive surgery, or multiple 6-mg/cycle doses postoperatively, with/without myelosuppressive chemotherapy, was consistent with the clinical pharmacology and safety profile of reference pegfilgrastim.

**Trial registration:**

October 2017. ClinicalTrials.gov Identifier: NCT02650193. EudraCT Number: 2015-002057-35.

**Supplementary Information:**

The online version contains supplementary material available at 10.1007/s00280-021-04355-6.

## Introduction

Myelosuppressive chemotherapy is a clinically important iatrogenic cause of febrile neutropenia, with chemotherapy-induced complications of neutropenia largely contributing to dose-limiting toxicity (DLT) [1]. Severe neutropenia and febrile neutropenia can prevent the completion of targeted therapy via delays in treatment, dose reductions, and discontinuations of chemotherapy. DLT can also cause a patient to be unable to complete chemotherapy without undue Grade 3 or 4 therapeutic toxicity [2, 3], potentially compromising long-term survival.

In addition to the myelotoxicity of many chemotherapeutic regimens, patient risk factors for myelosuppression (age > 65 years, persistent neutropenia, liver or renal dysfunction) and disease characteristics (bone, bladder, and pancreatic cancers) can affect the risk of developing febrile neutropenia [4–6]. The prophylactic use of granulocyte colony-stimulating factors (G-CSF) is therefore recommended for patients at high (> 20%) or intermediate (10–20%) risk of febrile neutropenia to reduce the risk of severe, potentially life-threatening infections and hospitalization [1, 7–10].

Endogenous G-CSFs are the primary cytokines regulating the activation, proliferation, differentiation, maturation, and survival of neutrophil precursor cells in bone marrow, as well as mature neutrophil cell function. During antineoplastic therapy (chemotherapy, radiotherapy, or chemoradiotherapy), endogenous G-CSF can be inadequate to counteract myelosuppression [11]. In the context of chemotherapy, a neutrophil nadir is often reached within 7 days post-administration [12, 13]. Limiting nadir depth and duration can help minimize the development and incidence of subsequent neutropenic complications and limit chemotherapy dose reductions or delays [14].

PF-06881894 (pegfilgrastim-apgf; Nyvepria™, Pfizer, NY, USA), a pegylated version of endogenous G-CSF, has been approved by the US Food and Drug Administration (FDA), Health Canada, and European Medicines Agency as a biosimilar to reference pegfilgrastim (Neulasta®, Amgen Inc, Thousand Oaks, CA, USA) [15, 16]. Biosimilars are versions of already licensed reference medicines, with highly similar physicochemical and biological characteristics and no clinically meaningful differences in terms of safety, purity, and potency [17]. Pegfilgrastim-apgf treatment is indicated to decrease the incidence of infection, as manifested by febrile neutropenia, in patients with non-myeloid cancers who receive myelosuppressive anticancer drugs associated with a clinically significant incidence of febrile neutropenia [16]. PEGylation, the addition of polyethylene glycol (PEG) to a protein, prolongs the circulating half-life compared with the non-pegylated protein. PEGylation for pegfilgrastim-apgf consists of the addition of a 20 kDa monomethoxy-polyethylene glycol polymer moiety [18]. As a result, the required G-CSF dosing schedule may be decreased from once-daily filgrastim to once-per-chemotherapy cycle pegfilgrastim. This simplifies G-CSF use and supports achievement of target dose intensity of chemotherapy [11, 14, 19]. This simplification has supported increased patient access and higher adherence for pegfilgrastim over non-pegylated filgrastim [14, 20].

**Fig. 1:**
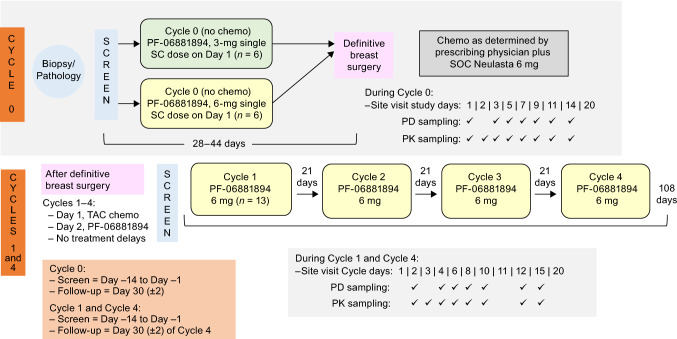
Study design. Chemo, chemotherapy; PD, pharmacodynamics; PK, pharmacokinetics; SC, subcutaneous; SOC, standard-of-care; TAC, docetaxel, doxorubicin and cyclophosphamide chemotherapy

This Phase I/II ascending-dose study was designed to assess the pharmacodynamics (PD), pharmacokinetics (PK), and safety (including immunogenicity) of PF-06881894 (Fig. 1), in development as a biosimilar to reference product Neulasta®. Each study phase (Phase I, Cycle 0; Phase II, Cycles 1–4) consisted of two independent study populations, differentiated by timing of PF-06881894 administration. Subjects in Phase I received PF-06881894 without chemotherapy and prior to definitive breast cancer surgery, whereas those in Phase II received PF-06881894 and concomitant adjuvant chemotherapy after definitive surgery. The study reported here was designed to assess 12 patients who received multiple 6-mg subcutaneous (SC) doses of PF-06881894 (Cycles 1–4). The 6-mg dose was based on the approved dosage for the reference drug, Neulasta.

## Materials and methods

### Study design

Study C1221002 was a two-phase, open-label, non-comparative, parallel-group study (Phase I, Cycle 0 and Phase II, Cycles 1–4, each with independent populations). The study was conducted across several European sites (Hungary and Spain) in women with non-distantly metastatic (non-stage IV) breast cancer. Cycle 0 was conducted without chemotherapy, before patients underwent definitive cancer surgery. Cycles 1–4 were conducted after definitive cancer surgery and included treatment with concomitant adjuvant chemotherapy (Fig. 1).

The primary objective of Cycle 0 was to characterize the PD response to a single 3- or 6-mg SC dose of PF-06881894 in chemotherapy-naïve women with non-distantly metastatic breast cancer. The PD variables, absolute neutrophil count (ANC) and CD34^+^ count, were used to determine whether it would be appropriate to study multiple doses of 3 mg in the context of background chemotherapy. Secondary objectives during Cycle 0 were to characterize the PK and safety profile of a single 3- or 6-mg SC dose.

The primary objective of Cycle 1 was to characterize the PD response of duration of severe Grade 4 neutropenia (DSN) to PF-06881894 when administered as single or multiple SC doses. Secondary objectives were to characterize the PD response (ANC) and PK profile of PF-06881894 in Cycles 1 and 4 when administered as single or multiple 6-mg SC doses, and to characterize the safety (including immunogenicity) of PF-06881894 when administered as single or multiple SC doses during Cycles 1–4.

This study was conducted in compliance with the protocol (ClinicalTrials.gov, NCT02650193; EudraCT Number: 2015-002057-35); the ethical principles originating in or derived from the 1964 Declaration of Helsinki; and in accordance with all International Council for Harmonisation Good Clinical Practice Guidelines. The final protocol, any amendments, and informed consent documentation were reviewed and approved by the Independent Ethics Committees at each site participating in the study. The original protocol was amended twice (July and December 2015) [21].

### Treatments

#### Cycle 0

Cycle 0 assessed a single 3- or 6-mg SC dose of PF-06881894 after diagnostic biopsy and prior to definitive breast surgery and without concomitant or background chemotherapy. The period post-biopsy and pre-definitive surgery was generally limited to ≤ 44 days. This timeframe was based on a maximum of 14 days for screening, 20 days for study assessments, and day 30 (± 2 days) for a follow-up visit. A longer time period was allowed if needed.

A 14-day screening period was used to determine the eligibility of six patients for enrollment in the PF-06881894 single 3-mg dose cohort in Cycle 0. After a comprehensive safety assessment of these six evaluable patients revealed no contraindications for dose-escalation, six additional patients were sequentially screened for enrollment. A single 6-mg dose of PF-06881894 was administered on day 1. By day 30 (± 2), each patient completed the subsequent visits for PD, PK, and safety (including anti-drug antibodies [ADAs]) assessments before ending their participation in the study.

In Cycle 0, both 3- and 6-mg doses demonstrated a neutrophil response consistent with published weight-based dosing data for Neulasta; as expected, the 3-mg dose demonstrated lower PD and PK results than the 6-mg dose, and therefore, 3-mg was not initiated in Cycles 1–4.

After Cycle 0 was completed, a separate study cohort (Cycles 1–4) was enrolled to receive multiple 6-mg SC doses of PF-06881894.

#### Cycles 1−4

Cycles 1–4 of docetaxel, doxorubicin, and cyclophosphamide (TAC) chemotherapy started after definitive breast surgery, according to standard of local practice. Patients received a 6-mg SC dose of PF-06881894 on day 2 (at least 24 h after chemotherapy) of four consecutive 21-day cycles of myelosuppressive TAC chemotherapy delivered on day 1 of each cycle.

### Study population

Eligible patients included women ≥ 18 years old with histologically confirmed and documented invasive breast cancer without evidence of distant metastases (non-Stage IV). Patients had body mass index (BMI) 19–40 kg/m^2^; Eastern Cooperative Oncology Group performance status ≤ 2 at screening; adequate bone marrow, hepatic and renal function reserve; were candidates for adjuvant chemotherapy with a TAC-based regimen; and chemotherapy-naïve. Main exclusion criteria included: known human epidermal growth factor receptor 2-positive or triple-negative breast cancer; any malignancy other than breast cancer (except adequately treated squamous or basal cell carcinoma of the skin, or cervical carcinoma in situ) within 5 years of the study; chemotherapy other than that included in this study; neoadjuvant chemotherapy or radiation therapy within 4 weeks; prior bone marrow or stem cell transplantation; malignancy within 5 years; known sickle cell disease; severe persistent drug-induced myelosuppression; active infection; known hypersensitivity to docetaxel, polysorbate 80, or doxorubicin; and/or previous exposure to a G-CSF or a biosimilar G-CSF. Medically necessary medications taken at the time of study entry or throughout the study were permitted, with recommendation to exclude patients who received inhibitors and inducers of cytochrome P450 3A4 or 2D6 and/or P-glycoprotein or doxorubicin or trastuzumab, consistent with reference drug product prescribing recommendations. A complete list of inclusion and exclusion criteria is in the protocol [21].

### Primary and secondary PD and PK endpoints

#### Cycle 0

During Cycle 0, the primary endpoint was the PD measurement of area under the effect versus time curve (AUEC) for ANC, from the time of dose administration to 288 h after dose administration (AUEC_ANC_). Secondary PD variables were maximum effect for ANC (ANC_*E*_max_); time of maximum effect for ANC (ANC__Tmax_); AUEC for CD34^+^ (${\rm{AUEC}}_{{\rm{CD34}}^{+}}$); maximum effect for CD34^+^ count (CD34^+^_E_max_); time of maximum effect for CD34^+^ count (CD34^+^_*T*_max_); AUEC_ANC_ from zero to infinity (AUEC_ANCinf_); and ${\rm{AUEC}}_{{\rm{CD34}}^{+}}$ from zero to infinity (${\rm{AUEC}}_{{\rm{CD34}}^{+}{\rm{inf}}}$).

Cycle 0 primary PK variables were area under the serum pegfilgrastim versus time curve (AUC) from time zero to time infinity (AUC_inf_) and the maximum observed pegfilgrastim concentration (*C*_max_) in Cycle 0. Secondary PK variables were AUC to time of last measurable concentration (AUC_t_); time to maximum serum concentration (*T*_max_); elimination half-life (t_1/2_); elimination rate constant (λz); and apparent clearance (CL/F).

#### Cycles 1–4

The primary PD parameter was DSN, i.e., days with Grade 4 neutropenia ANC < 0.5×10^9^/L in Cycle 1. Secondary PD variables were: DSN in Cycle 4; ANC nadir; time of nadir; AUEC; AUEC_ANCinf_; incidence of febrile neutropenia (i.e., tympanic or axillary body temperature > 38.5 °C for > 1 h with ANC < 1.0×10^9^/L); incidence of severe Grade 4 neutropenia; and time to ANC recovery (the first day with ANC ≥ 2.0×10^9^/L after any day with ANC < 2.0×10^9^/L) in Cycles 1 and 4. Additional PD parameters were ANC_*E*_max_ and ANC_*T*_max_ in Cycle 1 and Cycle 4.

Primary PK variables were AUC_t_ and *C*_max_ in Cycles 1 and 4. Secondary variables were AUC_inf_; *T*_max_; *t*_1/2_; λz; and CL/F in Cycles 1 and 4.

### Other prespecified endpoints

Safety assessments included the number of patients with treatment-emergent adverse events (TEAEs), serious AEs (SAEs) and AEs of special interest (AESI), and clinical laboratory abnormalities, as well as vital signs, 12-lead electrocardiogram (ECG), and/or physical examination abnormalities of clinical significance.

All medications taken within 7 days or five half-lives of screening (whichever was longer) were documented. Exposure to concomitant medication used during the study and immunogenicity (positive anti-pegfilgrastim and anti-polyethylene glycol [anti-PEG] antibody status) were also documented.

### PD and PK assessments

For the PD analysis during Cycle 0, blood samples were collected for ANC and CD34^+^ count, within 1 h prior to dose administration on Day 1 Cycle 0 and at 48, 96, 144, 192, 240, and 312 h postdose. ANC samples were tested at a central clinical laboratory. Flow cytometry was used for the CD34^+^ count.

For the PK analysis during Cycle 0, blood samples were collected within 1 h prior to dose administration on day 1 and at 6, 12, 24, 48, 96, 144, 192, 240, and 312 h postdose. Validated enzyme-linked immunosorbent assay (ELISA) methodology was used to determine serum pegylated filgrastim concentrations using a double-antibody sandwich method with quantitation by absorbance (range, 100–5000 pg/mL) [22, 23].

During Cycles 1–4, the same timepoints for PD (ANC only) and PK blood sample collection and methodologies applied as for Cycle 0 for like parameters, except that collections started on day 2 of chemotherapy in Cycles 1 and 4 only. PD and PK parameters were calculated using Phoenix WinNonlin (v6.4) non-compartmental analysis.

### Safety evaluations

All patients who received at least one dose of PF-06881894 were included in the safety population. AEs were reported from the time of informed consent up to and including the follow-up visit (day 30 ± 2) in Cycles 0 and 4 (depending on study phase) or early discontinuation. AEs were coded using Medical Dictionary for Regulatory Activities v20.1, and causality was determined by investigator assessment. When the relationship of an AE to PF-06881894 was unavailable, it was assumed to be PF-06881894-related.

AESIs were prospectively defined according to the US and EU product labels for pegfilgrastim reference Neulasta [15, 24].

Blood samples were collected to assess hematology (a complete blood count with platelets) and clinical chemistry (to evaluate organ function, diseases and/or disorders; and immunogenicity). During Cycle 0, sample collection for these assessments was at screening, during the treatment period (hematology: days 3, 7, 14, and 20; clinical chemistry: days 3, 7, 11, and 20) and at the follow-up visit (day 30 ± 2). During Cycles 1 and 4, samples were collected at screening, during the treatment period (hematology: days 3, 6, 10, 11, 12, 15, and 20; clinical chemistry: days 3, 6, 10, 11, 12, and 20), and after Cycle 4 at the follow-up visit (Day 30 ± 2) or upon patient discontinuation. In addition, for Cycles 2 and 3, samples were to be obtained for hematology on days 3, 6, 10, 11, 12, 15, and 20.

Urinalysis was performed at screening and at the Cycle 0 and Cycle 4 final follow-up visits. Investigators assessed any abnormalities for clinical significance. Independent of investigator assessment of abnormalities, all laboratory findings were subsequently graded according to the Common Terminology Criteria for Adverse Events v4.03.

A physical examination and a 12-lead resting ECG were performed at screening and after dosing at the Cycle 0 and Cycle 4 final follow-up visits. Changes over time were summarized by cycle within dose cohorts. Vital signs were monitored at screening, at every visit within cycles and at the Cycle 0 and Cycle 4 follow-up visits, or upon patient’s discontinuation.

### Immunogenicity

Blood samples were collected to test for anti-pegfilgrastim antibodies and anti-PEG antibodies during Cycle 0 on day 1 prior to dosing, and on days 14 and 20. ADA testing occurred on day 2 prior to dosing, and on day 20 of Cycles 1 and 4.

The methodology for antibody detection (Online Resource Methods S1) has been described previously by Moosavi et al*.* [25].

### Data analysis

The anticipated overall study population was 24 patients (*n* ~ 12 per study phase). PD and PK values were summarized by treatment cycle within dose cohorts using descriptive statistics for two populations: the full analysis set (FAS) and FAS excluding participants who were confirmed positive for anti-pegfilgrastim antibodies.

Safety data were summarized for the safety population for Cycle 0 and Cycles 1–4 within dose cohorts using descriptive statistics. AEs were listed for patients with a positive anti-pegfilgrastim antibody test and/or a positive anti-PEG test.

## Results

### Patient disposition

This Phase I/II study was conducted at ten sites (three sites in Hungary and seven in Spain) between 21 December 2015 and 5 October 2017. Three sites in Spain received study drug, but did not enroll patients.

Among the 31 patients screened for study participation, 25 (80.6%) were enrolled in the study and 6 (19.4%) were screen failures. During Cycle 0, 12 patients received a single dose of PF-06881894 (3- or 6-mg; *n* = 6/dose level).

During Cycles 1–4, 13 patients received multiple doses of PF-06881894 (6 mg per cycle). The 3-mg dose was not tested beyond Cycle 0 (as prespecified by study protocol). All enrolled patients across all cycles completed their respective study phase. The FAS, as well as the PD, PK, and safety populations, comprised 25 patients. No patients tested positive for anti-pegfilgrastim antibodies at any time point; there was no need to report on adjusted analysis sets with excluded patients.

Demographic and baseline characteristics for the FAS are summarized in Online Resource Table S1. The mean age at enrollment was 59 years, 96% of patients were White, with mean BMI of 30.6 kg/m^2^. At least one medical condition was reported for all 25 patients, with no clinically meaningful differences between Cycle 0 and Cycles 1–4.

#### Prior/concomitant medications

All but one (8.3%) patient in Cycle 0 (3-mg cohort) had received prior medication; the most common were angiotensin-converting-enzyme (ACE) inhibitors, acetylsalicylic acid, and/or benzodiazepine derivatives. The most commonly used concomitant medications during Cycle 0 included benzodiazepine derivatives and ACE inhibitors.

During Cycles 1–4, prior use of one or more of the most commonly used medications (by > 50% of patients for each drug) included glucocorticoids, serotonin antagonists, H2-receptor antagonists, and chloropyramine (the latter administered for management of chemotherapy side effects prior to receiving PF-06881894). The most common concomitant medications during Cycles 1–4 were the same as those used prior to Cycles 1–4. Each of these medications was used by ≥ 70% of patients.

### PD and PK

#### Cycle 0

**Fig. 2:**
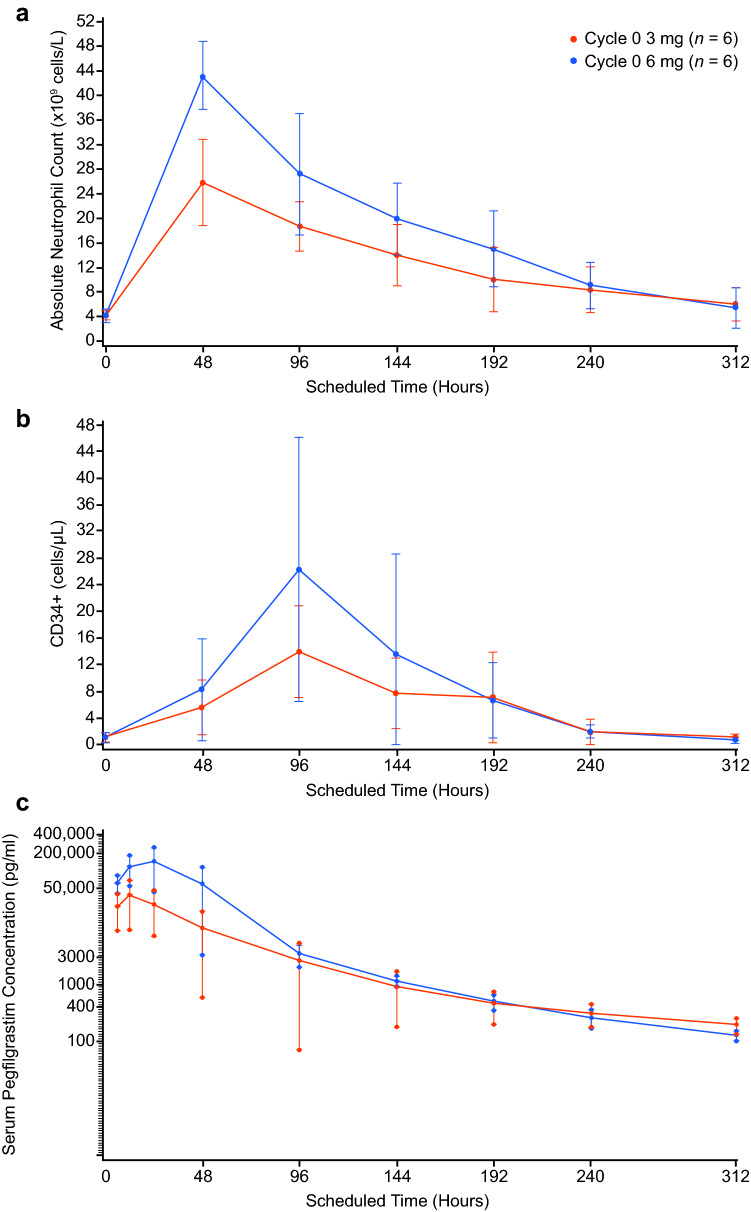
Pharmacological effects of a single ascending-dose of PF-06881894 over time in the absence of chemotherapy in Cycle 0. **a** ANC levels (FAS), **b** CD34^+^ (FAS), and **c** pegfilgrastim concentration (PK population). ANC, absolute neutrophil count; FAS, full analysis set; PK, pharmacokinetics

The mean ANC values and mean CD34^+^ count during the earlier time points assessed within Cycle 0 were consistently greater following the 6-mg dose than the 3-mg dose of PF-06881894 (Fig. 2a, b). The PK exposure parameters were also higher in the 6-mg versus 3-mg cohorts (AUC_t_ [h×pg/mL] mean ± SD: 5,677,700.3 ± 3,756,049.2 versus 1,410,202.6 ± 948,443.5 for the 6-mg versus 3-mg dose, respectively and C_*max*_ [pg/mL] mean ± SD: 155,766.7 ± 99,051.8 versus 38,026.7 ± 28,821.7 for the 6-mg versus 3-mg dose, respectively [Table 1]). Mean serum pegfilgrastim concentrations are shown by cohort in Fig. 2c.

**Table 1:** Summary of the pharmacological parameters in the single ascending-dose Cycle 0 (full analysis set)

Parameters	PF-06881894^a^	
	3 mg	6 mg
Subjects, *n*	6	6
Pharmacodynamics (ANC and CD34^+^)		
AUEC_ANC_ (h×10^9^/L)^b^	3900.482 (683.6870)	5880.985 (1287.2887)
ANC_*E*_max_ (×10^9^/L)^b^	24.512 (6.0710)	43.257 (5.5683)
ANC_*T*_max_ (h)^b^	71.95 [48.00–144.10]	47.80 [46.90–48.30]
${\rm{AUEC}}_{{\rm{CD34}}^{+}}$ (h×cells/μL)^b^	1749.523 (1022.3037)	2752.198 (2152.8794)
CD34^+^_*E*_max_ (cells/μL)^b^	13.970 (6.8536)	27.343 (18.4805)
CD34^+^_*T*_max_ (h)^b^	96.00 [48.00–96.10]	96.60 [95.80–191.30]
AUEC_ANCinf_ (h×10^9^/L)	5254.288 (1699.7088)^c^	6576.165 (1821.9919)
${\rm{AUEC}}_{{\rm{CD34}}^{+}{\rm{inf}}}$ (h×cells/μL)	1835.221 (1036.6473)	3159.470 (2197.4774)^c^
Pharmacokinetics (serum pegfilgrastim)		
AUC_t_ (h×pg/mL)^b^	1,410,202.6 (948,443.5)	5,677,700.3 (3,756,049.2)
* C*_max_ (pg/mL)^b^	38,026.7 (28,821.7)	155,766.7 (99,051.8)
AUC_inf_ (h×pg/mL)^b^	1,425,862.2 (949,518.8)	5,689,476.1 (3,757,035.5)
* T*_max_ (h)	12.0 [12–12]	23.5 [6–24]
* t*_1/2_ (h)^b^	50.0 (15.5)	48.8 (12.5)
λz (/h)^b^	0.015 (0.0051)	0.015 (0.0041)
CL/F (mL/h)^b^	4235.6 (4714.4)	1655.9 (1242.6)

^a^Data are mean (± standard deviation) or median [range]

^b^Three measurable values per subject, per parameter assessed, within a specific cohort or study cycle were needed for reliable calculation and inclusion in the results

^c^*n* = 5

*λz* elimination rate constant; *ANC* absolute neutrophil count; *ANC_E*_*max*_ maximum effect for ANC; *ANC_T*_*max*_ time of maximum effect for ANC; *AUEC*_*ANC*_ area under the effect versus time curve for ANC from the time of dose administration to 288 h after dose administration; *AUC*_*inf*_ area under the serum pegfilgrastim versus time curve from the time of dose administration to time infinity; *AUC*_*t*_ area under the serum pegfilgrastim versus time curve from the time of dose administration to the time of last measurable concentration; *AUEC*_*ANCinf*_ area under the effect versus time curve for ANC from the time of dose administration to time infinity; ${{AUEC}}_{{{CD34}}^{+}}$ area under the effect curve for CD34^+^; ${\rm{AUEC}}_{{\rm{CD34}}^{+}{\rm{inf}}}$ area under the effect curve for CD34^+^ from the time of dose administration to time infinity; *CD34*^+^*_E*_*max*_ maximum effect for CD34^+^ count; *CD34*^+^*_T*_*max*_ time of maximum effect for CD34^+^ count; *CL/F* apparent clearance, *C*_*max*_ maximum observed serum pegfilgrastim concentration; *t*_*1/2*_ elimination half-life; *T*_*max*_ time to maximum serum pegfilgrastim concentration

In the absence of myelosuppressive chemotherapy in Cycle 0, the 3-mg dose of PF-06881894 exhibited a less adequate and potentially subtherapeutic PD response relative to the 6-mg dose (lower AUEC_ANC_ [h×10^9^/L] mean ± SD: 3900.482 ± 683.6870 versus 5880.985 ± 1287.2887 for the 3-mg versus 6-mg dose, respectively and/or ${\rm{AUEC}}_{{\rm{CD34}}^{+}}$ [h×cells/μL] mean ± SD: 1749.523 ± 1022.3037 versus 2752.198 ± 2152.8794 for the 3-mg versus 6-mg dose, respectively [Table 1]). In addition, PK values were indicative of lower pegfilgrastim exposure at the 3-mg dose which was subsequently not included in Cycles 1–4.

#### Cycles 1–4

Severe Grade 4 neutropenia was observed in five (38.5%) patients; four of the same patients from Cycle 1 contributed to the count in Cycle 4 (Table 2).

**Table 2:** Summary of pharmacological parameters in the multiple-dose Cycle 1 and Cycle 4 (full analysis set)

Parameter	PF-06881894, 6-mg dose	
	Cycle 1	Cycle 4
Subjects, *n*	13	13
Pharmacodynamics^a^		
DSN (days)^b^	0.667 (0.9847)^c^	0.667 (0.9847)^c^
ANC nadir (×10^9^/L)	1.132 (1.1480)	1.623 (1.8364)
Time of ANC nadir (h)	129.231 (23.0585)	142.154 (65.3323)
AUEC_ANCt_ (h×10^9^/L)	2540.285 (854.2237)	3186.542 (1362.0079)
AUEC_ANCinf_ (h×10^9^/L)	5636.963 (1974.1635)^d^	12,399.370 (18,345.3366)^e^
ANC_*E*_max_ (×10^9^/L)^g^	18.286 (5.4720)	31.566 (12.3701)
ANC_*T*_max_ (h)^g^	47.80 [46.00–191.10]	47.90 [46.10–48.60]
Time to ANC recovery (days)^f^	2.615 (1.7097)	2.0 (1.633)
Pharmacokinetics (serum pegfilgrastim)^a^		
AUC_t_ (h×pg/mL)^g^	10,084,193.7 (14,047,222.7)	6,017,621.6 (5,920,395.4)
* C*_max_ (pg/mL)^g^	118,130.8 (119,028.6)	95,200.0 (93,544.1)
AUC_inf_ (h×pg/mL)^g^	10,093,213.5 (14,047,936.2)	6,425,013.3 (6,000,938.3)^c^
* T*_max_ (h)	24.1 [12–48]	23.5 [6–142]
* t*_1/2_ (h)^g^	30.7 (10.8)	29.5 (9.5)^c^
λz (/h)^g^	0.026 (0.0099)	0.025 (0.0060)^c^
CL/F (mL/h)^g^	1326.8 (1010.2)	2342.8 (2043.8)^c^

^a^Data are mean (± standard deviation) or median [range]

^b^DSN represents the days with severe Grade 4 neutropenia (ANC < 0.5 × 10^9^/L)

^c^*n* = 12

^d^*n* = 8

^e^*n* = 7

^f^Time to ANC recovery is the first day with ANC ≥ 2.0 × 10^9^/L after any day with ANC < 2.0 × 10^9^/L

^g^Three measurable values per subject, per parameter assessed, within a specific cohort or study cycle were needed for reliable calculation and inclusion in the results

*λz* elimination rate constant, *AUC*_*inf*_ area under the serum pegfilgrastim versus time curve from the time of dose administration to time infinity, *ANC* absolute neutrophil count, *ANC_E*_*max*_ maximum effect for ANC, *ANC_T*_*max*_ time of maximum effect for ANC, *AUC*_*t*_ area under the serum pegfilgrastim versus time curve from the time of dose administration to the time of last measurable concentration, *AUEC*_*ANCinf*_ area under the effect versus time curve for ANC from the time of dose administration to time infinity, *AUEC*_*ANCt*_ area under the effect versus time curve for ANC from the time of dose administration to the time of last measurable concentration, *CL/F* apparent clearance, *C*_*max*_ maximum observed serum pegfilgrastim concentration, *DSN* duration of severe Grade 4 neutropenia, *t*_*1/2*_ elimination half-life, *T*_*max*_ time to maximum serum pegfilgrastim concentration

The mean DSN for Cycle 1 and Cycle 4 were exactly the same (0.667 days); however, the PD response in Cycle 4 was comparatively more robust than in Cycle 1, exhibiting a higher and later ANC nadir, and recovery was more rapid (ANC nadir [×10^9^/L] mean ± SD: 1.623 ± 1.8364 versus 1.132 ± 1.1480 for Cycle 4 and 1, respectively, and time of nadir [h] mean ± SD: 142.154 ± 65.3323 versus 129.231 ± 23.0585 for Cycle 4 and 1, respectively [Table 2]).

**Fig. 3:**
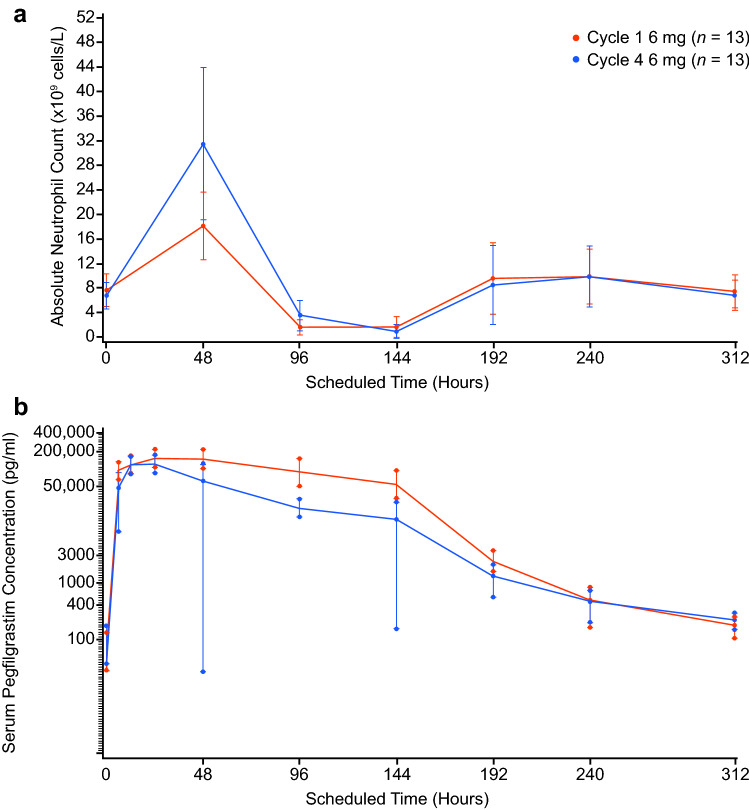
Pharmacological effects of multiple doses of PF-06881894 over time in the context of myelosuppressive chemotherapy in Cycles 1 and 4. **a** ANC levels (FAS) and **b** pegfilgrastim concentration (PK population). ANC, absolute neutrophil count; FAS, full analysis set; PK, pharmacokinetics

ANC levels over time for Cycles 1 and 4 are shown in Fig. 3. The ANC response was particularly robust within the initial 96 h of dose administration. Mean AUC and *C*_max_ values were lower and CL/F values were higher in Cycle 4 versus Cycle 1 (AUC_t_ [h×pg/mL] mean ± SD: 6,017,621.6 ± 5,920,395.4 versus 10,084,193.7 ± 14,047,222.7 for the 6-mg dose in Cycle 4 versus Cycle 1, respectively; C_max_ [pg/mL] mean ± SD: 95,200.0 ± 93,544.1 versus 118,130.8 ± 119,028.6 for the 6-mg dose in Cycle 4 versus Cycle 1, respectively; and CL/F [mL/h] mean ± SD: 2342.8 ± 2043.8 versus 1326.8 ± 1010.2 for the 6-mg dose in Cycle 4 versus Cycle 1, respectively [Table 2]).

### Safety

Overall, there were 161 (98.2%) TEAEs reported during this Phase I/II study. No patients had a TEAE that led to discontinuation of PF-06881894 or discontinuation from the study, and no deaths were reported. There were no injection-site reactions (ISRs) reported during Cycle 0 or Cycles 1–4.

#### Cycle 0

During Cycle 0, without chemotherapy, a total of 46 TEAEs were reported for all 12 (100%) patients, and the number of TEAEs was similar in the 3- and 6-mg single-dose cohorts (22 and 24 TEAEs, respectively) (Table 3). The most frequently reported TEAEs (> 2) were vertigo and backpain in the 3-mg dose cohort, and headache, backpain, and nausea in the 6-mg cohort. Most TEAEs of backpain and vertigo were treatment-related, and none were considered severe by the investigators (summarized in Online Resource Table S2). Two patients in the 6-mg PF-06881894 cohort experienced two events of dermatitis contact and leucocytosis (treatment-related), and both were mild in severity.

**Table 3:** Summary of the most frequent treatment-emergent adverse events (reported in > 2 subjects receiving any dose of PF-06881894 in either study phase per system organ class) in the safety population

	PF-06881894		
	Cycle 0 Single dose		Cycles 1–4
			Multiple doses
	3 mg	6 mg	6 mg
Subjects, *n*	6	6	13
Subjects who had TEAEs, *n* (%)	6 (100)	6 (100)	13 (100)
No. of TEAEs	22	24	115
Subjects who had treatment-related TEAEs, *n* (%)	5 (83.3)	6 (100)	5 (38.5)
No. of treatment-related TEAEs	11	12	12
Subjects who had treatment-emergent AESIs, *n* (%)	0	2 (33.3)^a^	2 (15.4)^b^
No. of treatment-emergent AESIs	0	2	3
Subjects who had a serious TEAE	0	0	2 (15.4)
No. of serious TEAEs	0	0	3^c^
All-causality TEAEs by system organ class, preferred term, *n* (%)			
Blood and lymphatic system disorders	0	1 (16.7)	4 (30.8)
Febrile neutropenia	–	–	2 (15.4)
Leukocytosis	0	1 (16.7)	–
Neutropenia	–	–	1 (7.7)
Thrombocytosis	–	–	1 (7.7)
Ear and labyrinth disorders	2 (33.3)	0	1 (7.7)
Vertigo	2 (33.3)	0	1 (7.7)
Gastrointestinal disorders	2 (33.3)	3 (50.0)	11 (84.6)
Abdominal distension	1 (16.7)	0	–
Abdominal pain	–	–	1 (7.7)
Abdominal pain upper	–	–	4 (30.8)
Aphthous ulcer	–	–	2 (15.4)
Constipation	–	–	1 (7.7)
Diarrhea	0	1 (16.7)	4 (30.8)
Dry mouth	–	–	1 (7.7)
Gingival pain	–	–	1 (7.7)
Hyperchlorhydria	–	–	1 (7.7)
Nausea	0	2 (33.3)	7 (53.8)
Vomiting	1 (16.7)	0	3 (23.1)
General disorders and administration-site conditions	2 (33.3)	0	5 (38.5)
Asthenia	–	–	2 (15.4)
Chest discomfort	1 (16.7)	0	–
Chills	–	–	1 (7.7)
Face edema	–	–	1 (7.7)
Fatigue	–	–	3 (23.1)
Inflammation	–	–	1 (7.7)
Pain	1 (16.7)	0	–
Pyrexia	–	–	2 (15.4)
Infections and infestations	1 (16.7)	1 (16.7)	3 (23.1)
Conjunctivitis	–	–	2 (15.4)
Nasopharyngitis	0	1 (16.7)	—
Skin infection	–	–	1 (7.7)
Viral infection	1 (16.7)	0	–
Musculoskeletal and connective tissue disorders	4 (66.7)	3 (50.0)	8 (61.5)
Back pain	2 (33.3)	2 (33.3)	4 (30.8)
Bone pain	–	–	2 (15.4)
Myalgia	–	–	3 (23.1)
Pain in extremity	1 (16.7)	1 (16.7)	3 (23.1)
Spinal pain	1 (16.7)	0	–
Nervous system disorders	2 (33.3)	5 (83.3)	6 (46.2)
Arachnoid cyst	1 (16.7)	0	–
Cerebral atrophy	0	1 (16.7)	–
Dizziness	–	–	1 (7.7)
Dysgeusia	–	–	1 (7.7)
Headache	1 (16.7)	4 (66.7)	5 (38.5)
Neuropathy peripheral	–	–	1 (7.7)
Skin and subcutaneous tissue disorders	0	1 (16.7)	11 (84.6)
Alopecia	–	–	8 (61.5)
Dermatitis contact	0	1 (16.7)	–
Erythema	0	1 (16.7)	3 (23.1)
Intertrigo	0	1 (16.7)	–
Pruritus	0	1 (16.7)	–
Vascular disorders	4 (66.7)	0	2 (15.4)
Arteriosclerosis	1 (16.7)	0	–
Flushing	1 (16.7)	0	–
Hot flush	–	–	1 (7.7)
Hypertension	1 (16.7)	0	1 (7.7)
Hypotension	1 (16.7)	0	–
Varicose vein	1 (16.7)	0	–

“Related” refers to any event that was assessed as either related or relationship is ‘missing’

^a^Events were Dermatitis contact from the category of Potential Allergic Reactions (1 event), and leucocytosis considered related to PF-06881894 (1 event), both non-serious and mild in nature

^b^Events were face edema from the category of Potential Allergic Reactions (one event) in one subject, which was considered mild in severity and not related to PF-06881894. Another subject had platelet count decreased from the category of Thrombocytopenia (two events based on change in severity; the first severe, the second moderate), both of which were considered not related to PF-06881894

^c^Events were febrile neutropenia, considered unrelated to PF-06881894, which were reported for two (15.4%) female subjects. Each woman was hospitalized following TAC chemotherapy (one event in both Cycle 1 and 2 for a 48-year-old subject; single event in Cycle 4 for a 64-year-old subject). These three events resolved with standard therapy (antibiotics and antipyretics)

*AESI* adverse event of special interest; *TEAE* treatment-emergent adverse event

No SAEs were reported during Cycle 0.

#### Cycle 1–4

During Cycles 1–4, a total of 115 TEAEs were reported among the 13 (100%) women, each of whom had at least one moderate or severe TEAE. The most frequently reported TEAEs (> 5) were alopecia, nausea, and headache. The most frequently reported treatment-related TEAEs are summarized in Online Resource Table S2.

A total of three SAEs of febrile neutropenia, considered unrelated to PF-06881894, were reported for two patients. Each woman was hospitalized following TAC chemotherapy (one event in both Cycle 1 and 2 for a 48-year-old patient; single event in Cycle 4 for a 64-year-old patient). These three events resolved with standard therapy (antibiotics, antipyretics). AESIs (three events: one face edema [of mild severity], two events of decreased platelet count in one patient) were reported in two patients, all were considered not related to treatment.

#### Clinical laboratory results

The clinical laboratory results and patterns observed were consistent with the known therapeutic response and safety profile for the US- and EU-approved pegfilgrastim (Neulasta). No patient in Cycle 0 or Cycles 1–4 had evidence of glomerulonephritis per urinalysis laboratory results. No changes in vital signs, ECG, and physical examination findings were considered clinically significant.

#### Immunogenicity

No patient tested for anti-pegfilgrastim antibodies was confirmed to be positive at any timepoint tested during Cycle 0 or Cycles 1 and 4 (Online Resource Table S3); therefore, no further characterization of the antibody response (e.g., neutralizing antibodies) was performed.

In contrast, anti-PEG antibodies were confirmed at one or more timepoints in all patients in the 3-mg dose group in Cycle 0 and none in Cycles 1 and 4. During Cycle 0, in the 3-mg dose cohort, one patient (16.7%) was anti-PEG-positive on day 1 prior to treatment. All six patients (100%) in the same cohort were anti-PEG-positive on day 14, and five (83.3%) were positive on day 20. At each corresponding time point in Cycle 0, two (33.3%) patients in the 6-mg single-dose phase were confirmed positive for anti-PEG. None of the 13 patients in Cycles 1 or 4 tested positive for anti-PEG antibodies at any study time point assessed.

No ISRs or AEs considered related to immunogenicity were reported, regardless of positivity for anti-PEG. After receiving a 3-mg dose of PF-06881894, one of the eight subjects who tested positive for anti-PEG antibodies had a non-serious AE of flushing that was mild in severity, deemed not related to PF-06881894, resolved within 24 h, and was considered to be attributable to a viral infection.

## Discussion

In this Phase I/II ascending-dose study, the PD, PK, and safety of single and multiple SC doses of PF-06881894 were assessed in women with non-distantly metastatic breast cancer. Initial development of PF-06881894 occurred during the early days of biosimilar development in the US, with evolution of requirements by the FDA during the trial. The trial design was adaptive to determine if a true dose escalation (3, 6, and 12 mg) would be required in the context of biosimilar development for the approved 6 mg dosing of the reference product.

The FDA agreed that assessment of the PD/PK/safety of 3- and 6-mg doses in patients without concomitant immunosuppressive chemotherapy would address first-in-human use of PF-06881894, without placing patients at unnecessary risk for inadequate PD (ANC) response. Additionally, concern for potential leukocytosis (> 100×10^9^/L) using a 12-mg dose led to a joint FDA-Sponsor decision regarding deferring of escalation to 12 mg until after assessment of results of Phase I (3 or 6 mg) and Phase II (6 mg only, if 3 mg was determined to be potentially subtherapeutic). After completion of all patients who received PF-06881894 in Cycle 0 it was agreed that it was appropriate not to study the 3-mg dose in patients receiving adjuvant chemotherapy, since that dose was deemed subtherapeutic relative to the 6-mg dose in Cycle 0 based on PD parameters. Additionally, it was jointly agreed with the FDA that escalation to the 12-mg dose would not be required to gain approval as a biosimilar.

When single-dose 3- or 6-mg PF-06881894 was administered to chemotherapy-naïve patients before definitive surgery (Phase I, Cycle 0), the neutrophil response was consistent with published, weight-adjusted dosing data for reference pegfilgrastim (Neulasta) [26], which indicated that both peak ANC level and duration of ANC response were dose-dependent.

The incidence of febrile neutropenia (~ 15%) observed in Cycles 1–4 is similar to other comparative studies of filgrastim biosimilars [27, 28]. In one study, the number proportion of patients reporting at least one episode of febrile neutropenia was only 9% (*n* = 12/139) for pegfilgrastim and 16% (*n* = 21/134) for biosimilar filgrastim [27]. Similarly, another study reported 9 events (*n* = 153) and 13 events (*n* = 248) of febrile neutropenia in patients taking prophylactic filgrastim for 5 and 7/10 days, respectively [28].

Based on the dose–response curve in patients with breast cancer [29], the average concentration (*C*_av_) of pegfilgrastim (calculated from the time of administration to the time of ANC nadir) for the 6-mg dose was 72 ng/mL, which corresponds to 90% of maximum effective concentration (EC_90_) response. For a 3-mg dose, the estimated *C*_av_ was ~ 21 ng/mL, which corresponds to ~ EC_70_. The expected response for a 3-mg dose (EC_70_) is 77.8% of that from a 6-mg dose (EC_90_). It is expected that ~ 30% of patients who received 3 mg pegfilgrastim would have a *C*_av_ value below the EC_50_ value. Therefore, a 3-mg dose of PF-06881894 may be potentially subtherapeutic in patients with breast cancer treated with myelosuppressive chemotherapy. The results in Cycle 0 confirmed the PD and PK of a 3-mg dose were less robust than those of a 6-mg dose. The lower systemic exposure to PF-06881894 in the 3-mg cohort is consistent with the lower ANC results (AUEC and *E*_max_) observed in this cohort. There were also differences in marrow response between the 3-mg and 6-mg cohorts based on the evidence provided by CD34^+^ counts. As a result of these findings and the safety risks of administration of a potentially subtherapeutic dose to patients receiving a chemotherapeutic regimen associated with clinically significant risk of myelosuppression and febrile neutropenia, the 3-mg dose was not included in Phase II (Cycles 1–4).

The DSN in patients receiving myelosuppressive chemotherapy is important in determining the risk for neutropenic fever and associated complications [30–32]. After recuperating from definitive surgery, the PK profile and PD response to a 6-mg/cycle regimen of PF-06881894 concomitant with chemotherapy were assessed during Cycles 1 and 4 to address any potential decrement in PD response over time. The observed PD response of < 1 day for DSN during both Cycles 1 and 4 is consistent with that reported in the literature for reference pegfilgrastim [33, 34]. The overall more robust PD response in Cycle 4 versus Cycle 1 of the Phase II study is also in line with reported data for reference pegfilgrastim [33, 34]. Liang et al. report that even with a larger difference of ± 40% in the ANC-AUEC between G-CSF products, the predicted mean difference in DSN between the products is still within ± 1 day [35]. The same result is observed when we compare the ANC-AUEC and DSN in our study to those in Liang et al.*,* the large difference of ANC-AUEC between two studies (our Cycle 1 versus Liang et al.: ~ 105 versus 71.6 or ~ 105 versus 70.5) is observed; however, the difference in DSN is still within 1 day (0.667 versus 1.1 day).

Neutrophil-mediated clearance is an important determinant of the physiologic response to pegfilgrastim [18, 33]. The higher ANC response (AUEC and E_max_), the lower AUC and *C*_max_ and the higher CL/F values of pegfilgrastim were observed in Cycle 4, when compared with corresponding values in Cycle 1. These are due to the neutrophil-mediated clearance of pegfilgrastim [18, 33] and higher ANC response in Cycle 4.

Anti-pegfilgrastim antibodies were not confirmed for any patient at any point during the Phase I/II study. Despite receiving multiple 6-mg doses of PF-06881894, patients in Cycles 1–4 did not exhibit an immunogenic response in terms of either anti-pegfilgrastim or anti-PEG.

Interestingly, anti-PEG antibodies were only confirmed among patients in Phase I, i.e., soon after cancer diagnosis and prior to any potentially immunosuppressive cancer therapy (definitive surgery or chemotherapy). The lack of anti-PEG antibodies in patients in Phase II may be related to these patients being more immunocompromised than patients in Phase I. Specifically, it is hypothesized that the lack of confirmed anti-PEG antibodies in the Phase II cohort is multifactorial and due to more time elapsing after their original cancer diagnosis combined with treatment with immunosuppressive cancer therapy including both definitive surgery and TAC chemotherapy.

The presence of anti-PEG antibodies in individuals who have not previously received a pegylated drug product is not unanticipated. It is well known that the prevalence of PEGylated polymers in a wide range of consumer products is a key reason why anti-PEG antibodies can be detected in ~ 72% of the general population at any time and in some patients with cancer pre-treatment [36].

During the overall clinical development for PF-06881894 as a biosimilar, uses of multiple 6-mg doses were studied in healthy volunteers (NCT02629289) [25]. The observed immune response in the current study was considered consistent with the known ADA profile in these healthy volunteers, in terms of both anti-pegfilgrastim and anti-PEG responses.

Minimal effect of ADAs was observed on the PK/PD profile of PF-06881894. The presence of anti-PEG antibodies in all patients in the 3-mg cohort in Cycle 0 precluded any assessment of their effect on PD or PK; however, it was noted that in the 6-mg cohort during Cycle 0, the two (33.3%) of six subjects who were positive for anti-PEG antibodies had the highest AUEC_ANC_ and the lowest AUC, likely attributable to a higher neutrophil-mediated clearance as opposed to an effect of anti-PEG antibodies on PK.

The results for platelet counts were consistent with those for US- and EU-pegfilgrastim reference products. Results for PF-06881894 in healthy volunteers demonstrated return of counts to baseline levels by follow-up visit [25], and was also demonstrated in Phase II of this study. In addition, the observed clinical laboratory findings and safety profile were consistent with the known therapeutic response for the US- and EU-approved pegfilgrastim reference product (Neulasta) [25, 37].

This trial was originally designed as a dose-finding study, but was transitioned by the Sponsor to a biosimilar regulatory pathway. The Phase I/II study provided supportive data in patients with breast cancer for biosimilar submission, as agreed in discussions with the FDA. Despite the inherent strengths of this unique adaptive study design, used to avert a subtherapeutic or supratherapeutic dosing regimen for patients, it also has some potential limitations. The study was non-comparative in nature; however, equivalent clinical efficacy and safety between pegfilgrastim reference products and pegfilgrastim biosimilars have been demonstrated in Phase I and II studies [25, 38, 39] in healthy volunteers [40] and cancer patients receiving chemotherapy [37, 41–46]. Another possible limitation of this study is the relatively small number of patients in the study population, which may lead to some interpretation bias of results. The target-mediated drug elimination could not be evaluated in this study.

## Conclusions

The safety and efficacy of a single 3-mg or 6-mg dose of PF-06881894 without chemotherapy or a 6-mg/cycle dose over multiple chemotherapy cycles was consistent with the known safety profile and therapeutic response of Neulasta. Immunogenicity did not influence safety, and, overall, no new safety concerns emerged.

In Cycle 0, in the absence of chemotherapy for the treatment of breast cancer, results showed that while PD response (ANC and CD34^+^) to a 6-mg dose of PF-06881894 was robust, the 3-mg dose was potentially subtherapeutic.

The mean duration of chemotherapy-induced severe neutropenia (DSN) did not decline over time in women with breast cancer from Cycle 1 to Cycle 4 (assessed in Cycles 1 and 4). The DSN in Cycle 1 and Cycle 4 was consistent with that reported for pegfilgrastim in the literature.

## Supplementary Information

Below is the link to the electronic supplementary material.

Supplementary file1 (DOCX 21 kb)

## Data Availability

Upon request, and subject to certain criteria, conditions, and exceptions (see https://www.pfizer.com/science/clinical-trials/trial-data-and-results for more information), Pfizer will provide access to individual de-identified participant data from Pfizer-sponsored global interventional clinical studies conducted for medicines, vaccines, and medical devices: 1) for indications that have been approved in the USA and/or EU; or 2) in programs that have been terminated (i.e., development for all indications has been discontinued). Pfizer will also consider requests for the protocol, data dictionary, and statistical analysis plan. Data may be requested from Pfizer trials 24 months after study completion. The de-identified participant data will be made available to researchers whose proposals meet the research criteria and other conditions, and for which an exception does not apply, via a secure portal. To gain access, data requestors must enter into a data access agreement with Pfizer.
