# Supplementary material for: Phase I/II study to assess the clinical pharmacology and safety of single ascending and multiple subcutaneous doses of PF-06881894 in women with non-distantly metastatic breast cancer
Source: Cancer Chemother Pharmacol. 2021 Oct 7;88(6):1033–48. doi: 10.1007/s00280-021-04355-6 (PMC8536579; doi:10.1007/s00280-021-04355-6)
Supplement: Supplementary file 1 — Supplementary file1 (DOCX 21 kb) [file 280_2021_4355_MOESM1_ESM.docx]

**Online Resource S1**

In brief, it involved the use of a validated electrochemiluminescent (ECL) assay format to detect the presence of anti-pegfilgrastim antibodies and a validated ELISA method was used for the analysis of anti-PEG antibodies. Samples confirmed positive for anti-pegfilgrastim antibodies underwent further characterization for filgrastim specificity and antibody titre via the same ECL assay. Samples confirmed as positive for anti-PEG antibodies were characterized for antibody titre using ELISA assay format. The ADA screening assay cut-point factor was 1.13 using a 95% confidence interval (CI). The confirmatory assay cut-point was 19.2% using 99% CI. A validated cell-based method was to be employed to assess samples confirmed as positive for neutralizing antibodies.

**Online Resource Table S1** Summary of demographic and baseline characteristics

|  | **PF-06881894** | | |  |
| --- | --- | --- | --- | --- |
|  | **Cycle 0** | | **Cycles 1–4** | **Total** |
|  | **3 mg** | **6 mg** | **6 mg** |  |
| Age (years)^a^ |  |  |  |  |
| Mean (SD) | 66.8 (8.9) | 60.8 (13.6) | 55.1 (9.0) | 59.3 (10.9) |
| Median | 64.5 | 64.5 | 53.0 | 61.0 |
| Range | 57.0–78.0 | 40.0–78.0 | 39.0-67.0 | 39.0-78.0 |
| Race, *n* (%) |  |  |  |  |
| Asian | 1 (16.7) | 0 | 0 | 1 (4.0) |
| White | 5 (83.3) | 6 (100) | 13 (100) | 24 (96.0) |
| Gender, *n* (%) |  |  |  |  |
| Female | 6 (100) | 6 (100) | 13 (100) | 25 (100) |
| Ethnicity, *n* (%) |  |  |  |  |
| Non-Hispanic/Latino | 6 (100) | 5 (83.3) | 10 (76.9) | 21 (84.0) |
| Not reported | 0 | 1 (16.7) | 2 (15.4) | 3 (12.0) |
| Unknown | 0 | 0 | 1 (7.7) | 1 (4.0) |
| Height (cm) |  |  |  |  |
| Median | 160.2 (5.5) | 154.2 (8.6) | 161.2 (6.7) | 159.2 (7.3) |
| Range | 151.0–167.0 | 142.0–168.0 | 152.0–175.0 | 142.0–175.0 |
| Weight (kg) |  |  |  |  |
| Mean (SD) | 80.4 (20.6) | 80.1 (12.2) | 74.7 (12.3) | 77.4 (14.3) |
| Range | 56.0–108.0 | 61.9–93.7 | 53.0–95.0 | 53.0–108.0 |
| Body mass index (kg/m^2^) |  |  |  |  |
| Mean (SD) | 31.2 (7.1) | 33.8 (2.6) | 28.9 (5.1) | 30.6 (5.4) |
| Range | 22.0–39.0 | 31.0–38.0 | 21.0–36.0 | 21.0–39.0 |

^a^Age is the integer value of (date of consent ─ date of birth) / 365.25 in years.

*SD* standard deviation.

**Online Resource Table S2** Summary of the most frequent treatment-related treatment-emergent adverse events (reported in >2 subjects receiving any dose of PF-06881894 in either study phase per system organ class) in the safety population

|  | **PF-06881894** | | |
| --- | --- | --- | --- |
|  | **Cycle 0** | | **Cycles 1–4** |
|  | **Single dose** | | **Multiple dose** |
|  | **3 mg** | **6 mg** | **6 mg** |
| Subjects, *n* | 6 | 6 | 13 |
| Subjects who had TEAEs, *n* (%) | 6 (100) | 6 (100) | 13 (100) |
| **Treatment-related TEAEs by system organ class, preferred, *n* (%)** | | | |
| Gastrointestinal disorders | 1 (16.7) | 1 (16.7) | 3 (23.1) |
| Abdominal pain upper | 0 | 0 | 1 (7.7) |
| Aphthous ulcer | 0 | 0 | 1 (7.7) |
| Nausea | 0 | 1 (16.7) | 1 (7.7)) |
| Vomiting | 1 (16.7) | 0 | 0 |
| Musculoskeletal and connective tissue disorders | 3 (50.0) | 2 (33.3) | 4 (30.8) |
| Back pain | 1 (16.7) | 2 (33.3) | 1 (7.7) |
| Myalgia | 0 | 0 | 3 (23.1) |
| Pain in extremity | 1 (16.7) | 0 | 0 |
| Spinal pain | 1 (16.7) | 0 | 0 |
| Nervous system disorders | 1 (16.7) | 3 (50.0) | 2 (15.4) |
| Headache | 1 (16.7) | 3 (50.0) | 2 (15.4) |

Adverse event terms were coded using MedDRA dictionary, version 20.1

*TEAE* treatment-emergent adverse event

**Online Resource Table S3** Anti-drug antibodies detected during Cycle 0 and Cycles 1 and 4 of the study (safety population)

|  |  | **PF-06881894** | |
| --- | --- | --- | --- |
| **ADAs confirmed as positive** | **Visit** | **Cycle 0** | |
|  |  | **Single dose**  **(*n*=6 per dose level)** | |
|  |  | **3 mg** | **6 mg** |
| Anti-pegfilgrastim | Day 1 | 0 | 0 |
|  | Day 14 | 0 | 0 |
|  | Day 20 | 0 | 0 |
|  | At any visit | 0 | 0 |
| Anti-PEG | Day 1 | 1 (16.7) | 0 |
|  | Day 14 | 6 (100.0) | 2 (33.3) |
|  | Day 20 | 5 (83.3) | 2 (33.3) |
|  | At any visit | 6 (100.0) | 2 (33.3) |
|  |  | **Cycles 1 and 4** | |
|  |  | **6-mg dose**  **(*n*=13 per cycle)** | |
|  | **Visit** | **Cycle 1**  **(after a single dose)** | **Cycle 4**  **(after multiple doses)** |
| Anti-pegfilgrastim | Day 2 | 0 | 0 |
|  | Day 20 | 0 | 0 |
|  | At any visit | 0 | 0 |
| Anti-PEG | Day 2 | 0 | 0 |
|  | Day 20 | 0 | 0 |
|  | At any visit | 0 | 0 |

*ADAs* anti-drug antibodies; *PEG* polyethylene glycol
